# The significance of equivocal bone findings in staging PSMA imaging in the preoperative setting: validation of the PSMA-RADS version 1.0

**DOI:** 10.1186/s13550-020-00745-8

**Published:** 2021-01-06

**Authors:** Jonathan Kuten, Snir Dekalo, Ishai Mintz, Ofer Yossepowitch, Roy Mano, Einat Even-Sapir

**Affiliations:** 1 Departments of Nuclear Medicine, Tel-Aviv Sourasky Medical Center, 6 Weizmann St, 6423906 Tel-Aviv, Israel; 2 Departments of Urology, Tel-Aviv Sourasky Medical Center, Tel Aviv, Israel; 3 Sackler School of Medicine, Tel-Aviv University, Tel-Aviv, Israel

**Keywords:** 68 Ga-PSMA, PET/CT, Prostate cancer, Equivocal, Bone metastases

## Abstract

**Background:**

Assessing the extent of disease in newly diagnosed prostate cancer (PC) patients is crucial for tailoring an appropriate treatment approach. Prostate-specific membrane antigen (PSMA)–targeted positron emission tomography/computed tomography (PET/CT) reportedly has greater accuracy than conventional imaging for staging PC. As with any imaging modality, pitfalls and nonspecific findings do occur. The PSMA reporting and data system (PSMA-RADS) version 1.0 offers structured interpretation of PSMA-targeted studies and classifies lesions by likelihood of clinical significance. The aim of this retrospective study was to evaluate the clinical significance of equivocal bone findings on staging PSMA-targeted imaging, as defined by PSMA-RADS version 1.0, in the preoperative setting. Fifteen of 406 consecutive patients staged by PET/CT prior to radical prostatectomy had equivocal bone lesions. The scans were retrospectively scored with the PSMA-RADS version 1.0 system, blinded to disease course and follow-up data. Postoperative persistence of prostate-specific antigen levels supported by imaging and histological findings was used as the reference standard for the true significance of equivocal imaging findings.

**Results:**

Thirteen of the 15 patients had an overall PSMA-RADS score of 3B, of whom only two had true metastatic disease. The remaining patients had scores of 4 (*n* = 1) or 5 (*n* = 1), all confirmed as true positive prostate-related malignant lesions. A per-lesion analysis identified 29 bone lesions, of which 27 were scored PSMA-RADS 3B, and only three of them were true metastases. Thus, debatable lesions proved to have no clinical significance in 84.6% of cases, and only 11% of equivocal PSMA-RADS 3B bone lesions were true positive.

**Conclusions:**

In intermediate and high-risk patients staged prior to radical prostatectomy, the majority of PSMA-RADS 3B lesions are of no clinical relevance. Bone lesions judged as being highly suspicious for metastases (PSMA-RADS 4/5) were all validated as true positives.

## Introduction

Prostate-specific membrane antigen (PSMA) is a type II transmembrane glycoprotein overexpressed on prostate cancer (PC) cells and serves for tumor-targeted imaging with positron emission tomography/computed tomography (PET/CT) [1]. PSMA-targeted PET/CT has been shown to be of high diagnostic value, outperforming conventional imaging both in the setting of biochemical failure and staging of intermediate and high-risk PC [2–16]. Several tracers are now available, namely 68 Ga- and 18F-labeled PSMA. While 68 Ga-PSMA-11 [68 Ga(HBED-CC)] is undisputedly the one most commonly used, 18F-labeled tracers, including the more recently introduced 18F-PSMA-1007, are gaining increased popularity, given their advantages of central cyclotron large-batch production and a longer half-life [17].

As with any sensitive imaging modality, equivocal findings are inevitable. These may warrant further workup, primarily to exclude the possibility of a false-positive finding [18–24]. It has been suggested that although 18F-PSMA-1007 PET/CT has equal or even superior detectability compared to 68 Ga-PSMA-11 [25–27], it may also be prone to more false-positive findings [28]. In the preoperative setting, for instance, a false-positive bone metastasis reading will modify the original treatment plan and unjustifiably exclude patients from receiving local definitive therapy with curative intent [29]. Data on the significance of these indeterminate lesions on staging PSMA-targeted imaging are lacking. Several systems have been proposed to facilitate the interpretation of PSMA-targeted PET scans [30–33]. These systems allow for better communication between nuclear physicians and referring clinicians and offer a standardized classification scheme of equivocal findings to guide further management. The PSMA-RADS version 1.0 reportedly has a low inter-observer variability, even among readers with varying levels of experience [34, 35], however, comparison to a reference standard is lacking.

We sought to evaluate the clinical significance of equivocal bone findings on PSMA-targeted imaging in the preoperative setting, as defined by PSMA-RADS version 1.0, and to compare it to the reference standard of undetectable post-radical prostatectomy (RP) prostate-specific antigen (PSA) levels.

## Materials and methods

### Patients

After obtaining institutional review board approval (663-20-TLV), we queried our prospectively maintained institutional database between 2015 and 2020 to retrieve the 406 registered consecutive PC patients classified as intermediate and high-risk according to the National Comprehensive Cancer Network (NCCN) risk stratification scheme [36]. All patients had undergone systemic staging by PSMA-targeted PET/CT in our medical center, followed by RP and pelvic lymph node dissection (PLND). Patients with PSMA-avid primary tumors and indeterminate bone findings were not uniformly declined the opportunity of surgery as a local treatment modality but rather counseled about the probability of false-positive PET findings versus the likelihood of genuine oligometastatic disease, with the latter mandating future systemic and/or bone-targeted therapy. Prostatic lesions, pelvic PSMA-positive LNs, as well as lesions considered benign (PSMA-RADS 1 and 2) were not considered contraindicators of surgery as the definitive treatment option.

### Imaging protocol

PET/CT studies were performed with either the Discovery 690 or the Discovery MI PET/CT systems (GE Healthcare) as previously described in depth elsewhere [1, 17, 37]. Patients were properly hydrated, asked to void immediately before acquisition, and scanned from mid-thigh to the tip of the skull approximately 60 min after 68 Ga-PSMA-11 at a dose of 1.8–2.2 MBq per kilogram bodyweight was injected intravenously or approximately 90 min after 18F-PSMA-1007 after a dose of 4 MBq per kilogram bodyweight was injected intravenously. A diagnostic CT scan was acquired by means of automatic mA-modulation, and 120 kV. CT data were used for PET attenuation correction.

PET images were acquired in a three-dimensional (3D) mode with an acquisition time of 3 min per bed position, and a matrix size of 128 × 128 (Discovery 690) or 256 × 256 (Discovery MI), and reconstructed with the VUE Point FX method by GE Healthcare that uses time of flight information and includes a fully 3D-ordered subset expectation maximization algorithm with 3 iterations/24 subsets and a filter cutoff of 8.0 mm (Discovery 690, 68 Ga), 2 iterations/24 subsets and a filter cutoff of 6.4 mm (Discovery 690, 18F), 3 iterations/8 subsets and a filter cutoff of 6.0 mm (Discovery MI, 68 Ga), or 4 iterations/8 subsets and a filter cutoff of 6.0 mm (Discovery MI, 18F), as well as corrections for normalization, attenuation, scatter, randoms and dead time. A standard Z-filter was applied to smooth between transaxial slices.

### Image analysis

Staging PSMA PET/CT scans were re-reviewed by a dedicated nuclear medicine specialist experienced in interpreting PSMA studies (JK), who was blinded to all other clinical and otherwise available imaging data, including postoperative PSA levels. Individual bone lesions were categorized by means of the PSMA reporting and data system (PSMA-RADS) version 1.0, and an overall score was derived based on the highest individual lesion score (excluding the primary prostatic lesion and pelvic lymph nodes).

## Reference standard

Postoperative PSA measurements, correlative imaging modalities, follow-up PSMA-targeted scans, and histopathological biopsy specimens, when available, were used as reference standards to establish whether lesions suspicious on imaging were clinically relevant. Persistence of PSA levels was defined as a post-RP PSA of ≥ 0.1 ng/mL [38].

### Statistical analysis

Descriptive statistics were performed using Microsoft Excel (2018).

## Results

Fifteen of the 406 patients (3.7%) with equivocal PET/CT findings were identified. Thirteen of these 15 had an overall PSMA-RADS 3B score (equivocal bone lesions), and two had bone lesions classified as PSMA-RADS 4 and 5, the latter highly suggestive of metastatic disease. Detailed information on patient characteristics, PSA levels, imaging findings, and correlative data are presented in Table 1. None of the patients in the present study had equivocal soft-tissue lesions (PSMA-RADS 3A).

**Table 1:** Patient characteristics, overall PSMA-RADS scores, and correlative data (*n* = 15)

No	Age (y)	Biopsy ISUP	Pre-RP PSA	Clinical stage		Risk-group^a^	RP ISUP	EPE	SVI	Lymph nodes dissected/positive	Tracer	Overall PSMA-RADS	1st PSA (ng/ml)	2nd PSA (ng/ml)	Correlative modalities
1	62	2	5.5	T1c		1	2	n	n	7/0	18F-PSMA-1007	3B	0.04	0.07	MRI, CT
2	69	2	5.1		T2b	1	2	y	y	9/0	68 Ga-PSMA-11	3B	0	0	Rib cage X-ray
3	76	3	15		T1c	2	3	y	y	13/0	68 Ga-PSMA-11	4	1.4	n/a^b^	MRI, follow-up PSMA PET/CT
4	74	3	5		T1c	2	3	y	n	8/0	68 Ga-PSMA-11	3B	0.09	0.09	None
5	64	2	4.5		T1c	3	2	n	n	12/0	68 Ga-PSMA-11	3B	0.09	0.09	Follow-up PSMA PET/CT
6	73	3	16		T2a	2	2	Y	n	9/0	18F-PSMA-1007	3B	0	0	None
7	57	1	10.5		T2a	1	2	n	n	20/0	18F-PSMA-1007	3B	0	0	MRI
8	56	2	12.13		T2b	2	2	y	n	21/0	68 Ga-PSMA-11	3B	0	0	MRI^c^
9	69	3	10		T2b	2	3	y	n	19/0	68 Ga-PSMA-11	3B	0.54	0.81	MRI, follow-up PSMA PET/CT
10	46	2	13		T1c	2	2	y	n	21/0	68 Ga-PSMA-11	3B	0	0	Histopathology
11	70	2	6.3		T1c	1	3	y	n	14/0	68 Ga-PSMA-11	3B	0	0	None
12	51	3	25		T3	3	3	y	y	14/1	68 Ga-PSMA-11	3B	2.9	4.1	MRI, follow-up PSMA PET/CT
13	72	4	6.5		T1c	3	4	y	n	7/1	68 Ga-PSMA-11	5	3	22	Follow-up PSMA PET/CT
14	74	3	9		T1c	2	3	n	n	14/0	68 Ga-PSMA-11	3B	0	0	None
15	63	2	7		T1c	1	3	y	n	17/0	68 Ga-PSMA-11	3B	0	n/a	Follow-up PSMA PET/CT

*RP* radical prostatectomy, *ISUP* International Society of Urological Pathology, *EPE* extraprostatic extension, *SVI* seminal vesicle invasion, *MRI* magnetic resonance imaging

^a^NCCN risk group: 1 = favorable intermediate; 2 = unfavorable intermediate; 3 = high

^b^Patient already started on systemic therapy with PSA reduction

^c^MRI findings inconclusive. However, a CT scan performed 10 years earlier showed the same finding, supporting a nonprostatic etiology

Eleven of the 15 patients (73.3%) reached an undetectable PSA level following surgery. Of the four patients (26.7%) with PSA persistence after surgery, two were classified as PSMA-RADS 3B and two as PSMAR-RADS 4 and 5. These lesions are described in Table 2. Subsequent imaging studies (PET PSMA and magnetic resonance imaging [MRI]) confirmed the diagnosis of bone metastases in these four patients and guided further therapy.

**Table 2:** Patients with persistence of PSA levels (*n* = 4)

Patient No	Overall PSMA-RADS score	Lesion 1: location, uptake, PSMA-RADS score, CT findings	Lesion 2: location, uptake, PSMA-RADS score, CT findings	Lesion 3: location, uptake, PSMA-RADS score, CT findings	1st PSA (ng/ml)
3	4	Sacrum, intense, 4, tiny sclerotic lesion^a^	Right ramus pubis, mild, 3B, nonspecific mild sclerosis^a^	4th left rib, mild, 3B, none	1.4
9	3B	C4 vertebra, mild, 3B, small lucent lesion^a^	Right ilium, mild, 3B, nonspecific mild sclerosis		0.54
12	3B	Left ilium, mild, 3B, none^a^			2.9
13	5	Right ilium, intense, 5, small lytic lesion^a^			3

^a^True positive lesion

**Fig. 1:**
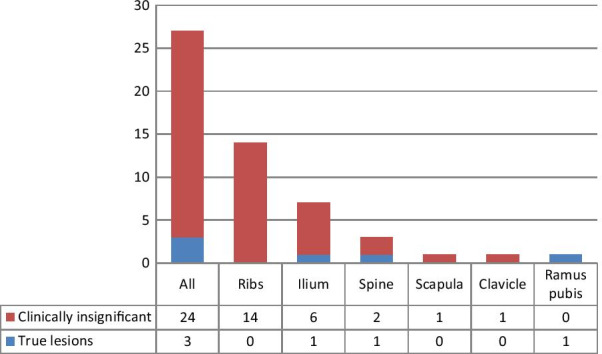
Bar plots of PSMA-RADS 3B lesions by location and clinical significance (*n* = 27)

**Fig. 2:**
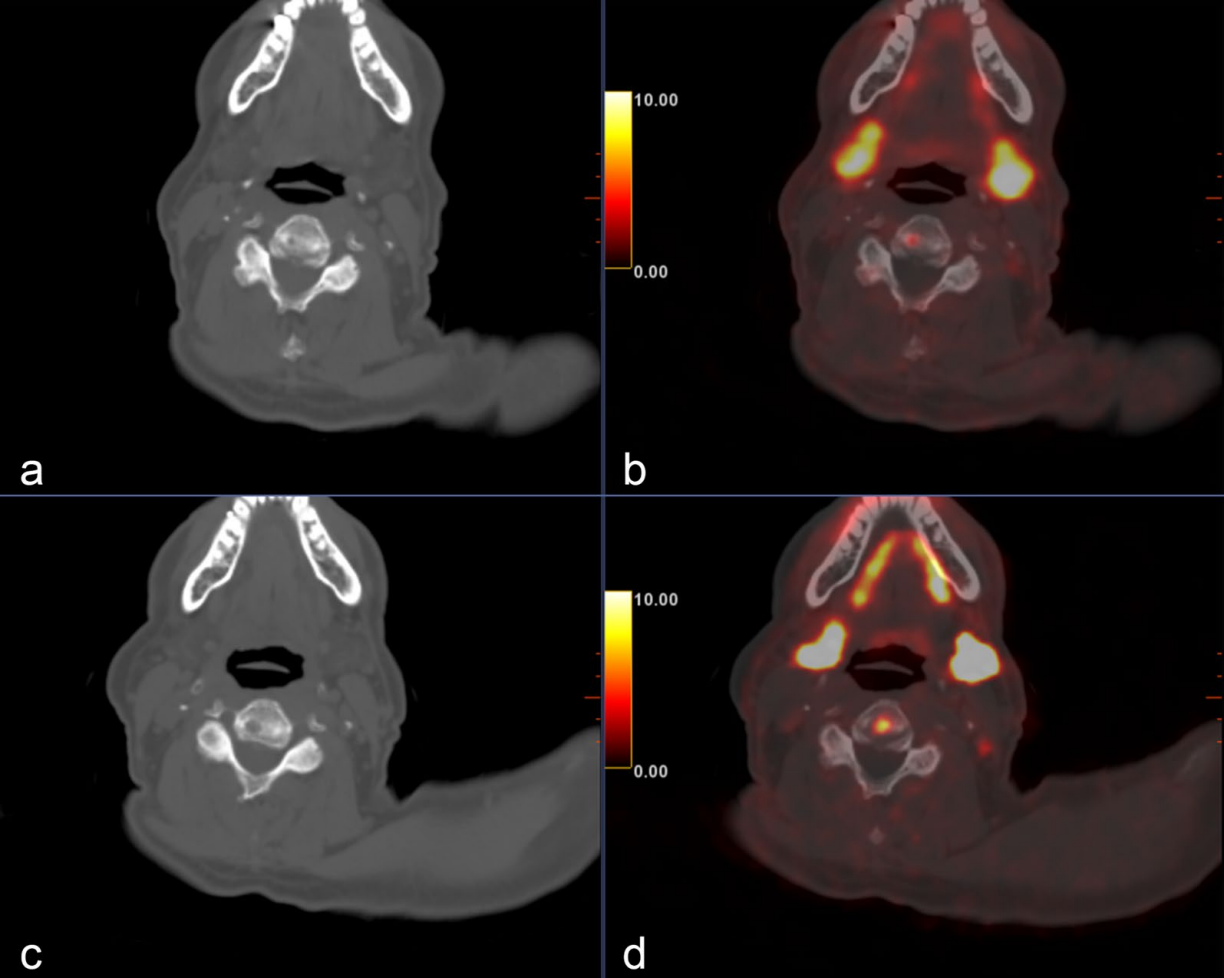
A 69-year-old patient (patient number 9) with unfavorable intermediate risk PC, who had PSA persistence after undergoing an RP. **a** Staging PET/CT, axial CT, and fused image showing a small lucent lesion in a C4 vertebral body with mild focal PSMA uptake, classified as PSMA-RADS 3B. **b** Postoperative PET/CT, axial CT, and fused image showing enlargement of the same lesion with more intense uptake. This patient had also a pelvic PSMA-RADS 3B lesion that remained unchanged. An MRI of the pelvis and spine supported these findings (not shown)

**Fig. 3:**
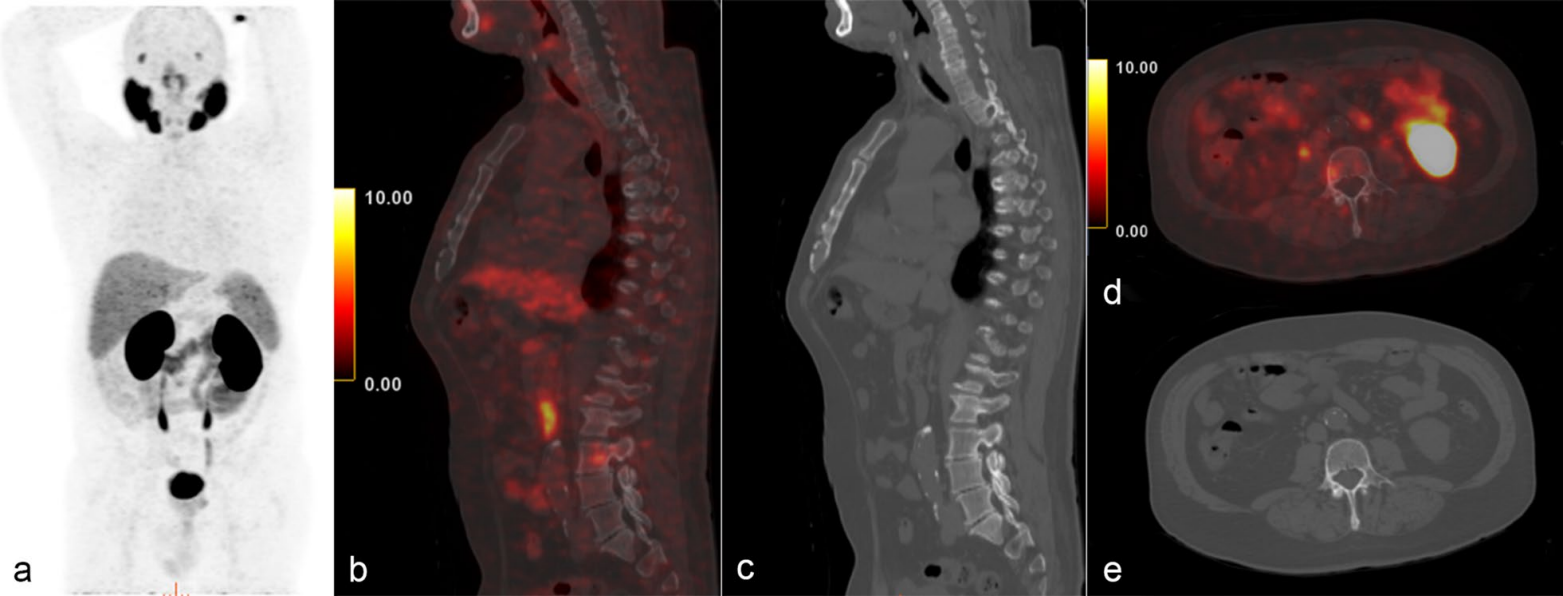
A 74-year-old patient (patient number 14) with unfavorable intermediate risk PC. Aside from the primary prostatic lesion, focal mild uptake in L3 vertebral body was identified on staging 68 Ga-PSMA-11 PET/CT, with no pathological findings on CT, categorized as a PSMA-RADS 3B lesion. This lesion was deemed as false-positive by Post-RP PSA measurements and clinical follow-up. Staging PET/CT mip (**a**); sagittal fusion (**b**) and CT (**c**); axial fusion (**d**) and CT (**e**)

A total of 29 bone lesions were studied in the 15 patients with apparently equivocal findings. Three of the 27 equivocal lesions (11.1%) that were reclassified as PSMA-RADS 3B were determined as being genuinely positive by means of postoperative PSA persistence and further confirmatory imaging data, whereas 24 were ultimately categorized as false-positive (Fig. 1). Figures 2 and 3 show examples of PSMA-RADS 3B lesions that were proven to be true positive and false positive, respectively. Twelve of these 15 patients underwent 68 Ga-PSMA-11 PET/CT and the other three underwent 18F-PSMA-1007 PET/CT. A total of 13 PSMA-RADS 3B lesions were detected in the latter subgroup, of which nine were in the ribs, and all were determined as being clinically insignificant (false-positive) by follow-up data. One patient with PSMA-RADS 3B lesions also had thickening of the terminal ileum and an enlarged mesenteric LN classified as PSMA-RADS 3D which was later diagnosed as a neuroendocrine tumor.

## Discussion

The skeletal system is the most common site of distant metastatic spread in PC [39]. Although PSMA-targeted imaging demonstrates improved sensitivity and specificity in detecting bone metastases compared to traditional imaging modalities, such as bone scintigraphy, CT, and MRI, in patients with primary intermediate or high-risk PC, PSMA-ligand uptake can appear in other benign as well as malignant osseous lesions [19, 23]. Data on the true nature of equivocal bone lesions on PSMA-targeted imaging are lacking.

In the present cohort of intermediate- and high-risk PC patients planned for RP, 15/406 (3.7%) patients had equivocal bone lesions on their preoperative PSMA-targeted PET/CT staging. Importantly, unlike previous studies, all of our patients underwent RP, after which PSA persistence is thought to be due to residual cancer [40]. This enabled the use of postoperative PSA test results as a highly sensitive reference standard for evaluating the true nature of findings on pre-surgical PSMA-targeted imaging. To the best of our knowledge, this is also the first study to utilize the PSMA-RADS version 1.0 in a cohort of patients who were evaluated for staging prior to RP.

The scans of 13 of our patients were classified as overall PSMA-RADS 3B, i.e., having equivocal bone lesions, and only two of them had evidence of true distant metastases after surgery. Thus, the debatable lesions proved to have no clinical significance in 84.6% of cases. On a per-lesion analysis, only 11% of equivocal PSMA-RADS 3B bone lesions were true positive. Furthermore, all of the lesions involving the ribs were deemed clinically insignificant.

Our current results are comparable to those of several previous studies. Yin et al. [41] evaluated equivocal findings on ^18^F-DCFPyL PET/CT and found that only a minority (21.4%) of PSMA-RADS 3B lesions were eventually diagnosed as true metastases. Unlike the current study that focused on patients presenting for staging prior to definitive surgery, their investigation was comprised of patients in different stages of disease and only follow-up PET was used as reference standard. Rauscher et al. [28] compared the frequency of PSMA-ligand-positive benign lesions between 68 Ga-PSMA-11 and 18F-PSMA-1007 PET/CT performed for biochemical failure, and those authors found that 24% and 27%, respectively, of the bone lesions to be false-positive. Those authors also showed that 18F-PSMA-1007 was prone to false-positive uptake in bone, especially in the ribs, and those results were supported by other publications [42] and consistent with those of our current study, in which the few patients who underwent 18F-PSMA-1007 PET showed more clinically insignificant nonspecific bone lesions.

Our findings support the concept that lesions classified as PSMA-RADS 3B are genuinely equivocal. However, in the setting of staging patients otherwise considered for RP, one may be reassured that the majority of patients with such lesions do not actually harbor metastatic disease, since these lesions will prove to be of no clinical significance, and, therefore, surgery should not be denied outright. Furthermore, the equivocal rib lesions, a well-recognized nonspecific finding on PSMA imaging, were all insignificant in the present patient population. Nevertheless, as suggested by the PSMA-RADS system, clinicians should be aware of the possibility of 3B lesions harboring malignancy, however remote and should consider follow-up of these indeterminate lesions or further imaging (i.e., MRI).

Two of our patients had lesions considered as being highly suggestive for PC metastases, and both proved to be true positive lesions by our reference standard. Another patient had lesions highly suspicious for non-prostatic malignancy, and that finding was validated by histopathology. Thus, the positive predictive values for PSMA-RADS scores 4, 5, and 3D were 100%. We recognize that this perfect predictive value is probably an overestimation due to the small number of patients in the present investigation and that larger trials are needed to further validate these findings.

Given the increasing use of PSMA-targeted imaging, not only for evaluating biochemical failure but also for staging, clinicians are faced with real-life dilemmas regarding the correct approach to not-uncommon equivocal findings on PSMA imaging. Our study group, however small, provides evidence-based answers for the multidisciplinary teams who are dealing with the need to decide upon the appropriate therapeutic management for this ever-growing group of patients.

Although the blinded retrospective interpretation of scans using a standardized reporting system that we applied in the current study, should have reduced potential biases, there are several limitations to this study, aside from the small number of patients and its retrospective nature. First, there is an inherent selection bias, since patients being considered for surgery with curative intent may be expected to less frequently have distant lesions. Furthermore, patients who were denied of RP, perhaps on the basis of their staging PSMA scan, were not captured in the present cohort. Another limitation is the use of two different PSMA-targeted tracers (18F-PSMA-1007 and 68 Ga-PSMA-11), although we consider that the use of a standardized interpretation system should overcome this limitation. Also, as 18F-PSMA-1007 has been suggested to be prone to false-positive skeletal findings, using both tracers in the current cohort may have led to an overestimation of the true rate of 3B lesions in pre-surgical candidates and consequently to a lower percentage of true-positives. Finally, data on histopathological confirmation as a gold-standard reference are lacking. Nevertheless, we believe that post-prostatectomy PSA levels with follow-up PET scans, when needed, are currently the most sensitive reference standard available for validation of distant lesions, considering that percutaneous biopsies are technically challenging and associated with low yield [43] and that metastasectomy is impractical and, for that matter, not a viable option.

In conclusion, the present study on intermediate- and high-risk PC patients prior to RP investigated the clinical significance of equivocal bone findings on staging PSMA imaging, as defined by the PSMA-RADS version 1.0 reporting system. The results of this study demonstrate that the majority of PSMA-RADS 3B lesions are of no clinical relevance in this group of patients.

## Data Availability

The datasets used and/or analyzed during the current study are available from the corresponding author on reasonable request.

## Abbreviations

PC: Prostate cancer
PSMA: Prostate-specific membrane antigen
PET/CT: Positron emission tomography/computed tomography
PSMA-RADS: The PSMA reporting and data system; RP: radical prostatectomy
NCCN: National Comprehensive Cancer Network
PLND: Pelvic lymph node dissection
3D: Three-dimensional
MRI: Magnetic resonance imaging
